# Supplementary material for: Frontopolar theta oscillations link metacognition with prospective decision making
Source: Nat Commun. 2021 Jun 24;12:3943. doi: 10.1038/s41467-021-24197-3 (PMC8225860; doi:10.1038/s41467-021-24197-3)
Supplement: Supplementary file 2 — Reporting summary [file 41467_2021_24197_MOESM2_ESM.pdf]

nature research

Corresponding author(s): Alexander Soutschek

Last updated by author(s): Apr 23, 2021

Reporting Summary

Nature Research wishes to improve the reproducibility of the work that we publish. This form provides structure for consistency and transparency in reporting. For further information on Nature Research policies, see our Editorial Policies and the Editorial Policy Checklist.

Statistics

For all statistical analyses, confirm that the following items are present in the figure legend, table legend, main text, or Methods section.

n/a	Confirmed	
☐	☒	The exact sample size (*n*) for each experimental group/condition, given as a discrete number and unit of measurement
☐	☒	A statement on whether measurements were taken from distinct samples or whether the same sample was measured repeatedly
☐	☒	The statistical test(s) used AND whether they are one- or two-sided<br>*Only common tests should be described solely by name; describe more complex techniques in the Methods section.*
☐	☒	A description of all covariates tested
☐	☒	A description of any assumptions or corrections, such as tests of normality and adjustment for multiple comparisons
☐	☒	A full description of the statistical parameters including central tendency (e.g. means) or other basic estimates (e.g. regression coefficient) AND variation (e.g. standard deviation) or associated estimates of uncertainty (e.g. confidence intervals)
☐	☒	For null hypothesis testing, the test statistic (e.g. *F*, *t*, *r*) with confidence intervals, effect sizes, degrees of freedom and *P* value noted<br>*Give P values as exact values whenever suitable.*
☐	☒	For Bayesian analysis, information on the choice of priors and Markov chain Monte Carlo settings
☐	☒	For hierarchical and complex designs, identification of the appropriate level for tests and full reporting of outcomes
☒	☐	Estimates of effect sizes (e.g. Cohen's *d*, Pearson's *r*), indicating how they were calculated

Our web collection on statistics for biologists contains articles on many of the points above.

Software and code

Policy information about availability of computer code

Data collection	Matlab 2016, Cogent toolbox
Data analysis	Matlab 2016, R 3.6.1, lme4 1.1-23, hBayesDM 1.0.2, Simnibs 2.1

For manuscripts utilizing custom algorithms or software that are central to the research but not yet described in published literature, software must be made available to editors and reviewers. We strongly encourage code deposition in a community repository (e.g. GitHub). See the Nature Research guidelines for submitting code & software for further information.

Data

Policy information about availability of data

All manuscripts must include a data availability statement. This statement should provide the following information, where applicable:
- Accession codes, unique identifiers, or web links for publicly available datasets
- A list of figures that have associated raw data
- A description of any restrictions on data availability

The data that support the findings of this study are available on Open Science Framework (https://osf.io/m57yg/). Source data are provided with this paper.

Field-specific reporting

Behavioural & social sciences study design

All studies must disclose on these points even when the disclosure is negative.

Study description	The study followed a within-subject design where all participants received theta, gamma, and sham tACS while performing computer-based tasks measuring metacognition and precommitment.
Research sample	We recruited 38 participants from the student population in Zurich (Mage = 22.9 years, range = 19-31, 17 female). As is common practice in neuroscience research, the sample was not representative of the entire population of Switzerland, however we investigated basic neuro-cognitive processes and were not interested in inter-individual variation. It is not to be expected that the neural basis of metacognition differs qualitatively between social groups or age groups, such that our sample allows drawing conclusions regarding the causal role of the frontopolar cortex in metacognition.
Sampling strategy	The sample size was determined with a power analysis (alpha = 5%, two-tailed, power = 80%) based on the effect size in our previous tDCS study on precommitment (Soutschek et al., 2017, SCAN).
Data collection	Participants performed computer-based tasks (programmed in Matlab using the Cogent toolbox) while being stimulated with tACS. Participants were blind to the current stimulation frequency and the hypotheses of the experiment. Only the researchers (AS or MM) and the study participants were present during the experiment. The researcher interacting with the participants was blinded to the tACS condition, but not to the research hypothesis.
Timing	Data were collected in April 2019.
Data exclusions	Data from one subject were excluded because the subject terminated the experiment prematurely due to tACS-induced side effects.
Non-participation	No volunteers declined participation.
Randomization	As the study followed a within-subject design, subjects were not allocated to different groups.

Reporting for specific materials, systems and methods

We require information from authors about some types of materials, experimental systems and methods used in many studies. Here, indicate whether each material, system or method listed is relevant to your study. If you are not sure if a list item applies to your research, read the appropriate section before selecting a response.

Materials & experimental systems

n/a	Involved in the study	
☒	☐	Antibodies
☒	☐	Eukaryotic cell lines
☒	☐	Palaeontology and archaeology
☒	☐	Animals and other organisms
☐	☒	Human research participants
☒	☐	Clinical data
☒	☐	Dual use research of concern

Methods

n/a	Involved in the study	
☒	☐	ChIP-seq
☒	☐	Flow cytometry
☒	☐	MRI-based neuroimaging

Human research participants

Policy information about studies involving human research participants

Population characteristics	See above
Recruitment	Participants were recruited via the internal participant pool of the SNS lab at the University of Zurich. This participant pool mainly includes University students, such that the sample cannot be considered as representative for the general population. As explained above, our sample nevertheless allowed drawing conclusions regarding the neural basis of metacognition, given that the neural implementation of metacognition is unlikely to qualitatively differ between social groups.
Ethics oversight	The study was approved by the Cantonal ethics committee Zurich.

Note that full information on the approval of the study protocol must also be provided in the manuscript.

April 2020
